# Nanostructured Solid/Liquid Acid Catalysts for Glycerol Esterification: The Key to Convert Liability into Assets

**DOI:** 10.3390/nano14070615

**Published:** 2024-03-31

**Authors:** John Keogh, Patcharaporn Inrirai, Nancy Artioli, Haresh Manyar

**Affiliations:** 1 School of Chemistry and Chemical Engineering, Queen’s University Belfast, David-Keir Building, Stranmillis Road, Belfast BT9 5AG, UK; 2 Department of Civil, Environmental, Architectural Engineering and Mathematics, University of Brescia, Via Branze, 43, 25123 Brescia, Italy

**Keywords:** glycerol, biodiesel, biofuels, fuel additives, net zero, esterification, solid acid catalysts

## Abstract

Owing to the growing concerns about the dwindling fossil fuel reserves, increasing energy demand, and climate emergency, it is imperative to develop and deploy sustainable energy technologies to ensure future energy supply and to transition to the net-zero world. In this context, there is great potential in the biorefinery concept for supplying drop in biofuels in the form of biodiesel. Biodiesel as a fuel can certainly bridge the gap where electrification or the use of hydrogen is not feasible, for instance, in heavy vehicles and in the farm and marine transportation sectors. However, the biodiesel industry also generates a large amount of crude glycerol as the by-product. Due to the presence of several impurities, crude glycerol may not be a suitable feedstock for all high-value products derived from glycerol, but it fits well with glycerol esterification for producing glycerol acetins, which have numerous applications. This review critically looks at the processes using nanostructured solid/liquid acid catalysts for glycerol esterification, including the economic viability of the scale-up. The homogeneous catalysts reviewed herein include mineral acids and Brønsted acidic ionic liquids, such as SO_3_H-functionalized and heteropoly acid based ionic liquids. The heterogeneous catalysts reviewed herein include solid acid catalysts such as metal oxides, ion-exchange resins, zeolites, and supported heteropoly acid-based catalysts. Furthermore, the techno-economic analysis studies have shown the process to be highly profitable, confirming the viability of glycerol esterification as a potential tool for economic value addition to the biorefinery industry.

## 1. Introduction

### 1.1. Biorefinery Concept

**Figure 1:**
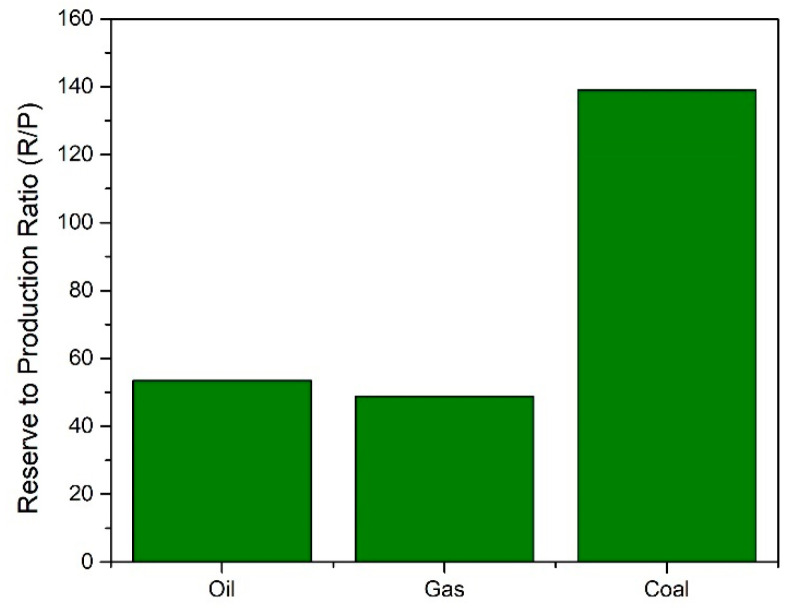
Fossil fuel reserve to production ratios [1].

Historically, the world has been reliant on conventionally used fossil fuels (oil, coal, and gas) for its supply of fuel, energy, and chemicals. This reliance, however, is not sustainable for several reasons. Firstly, fossil fuels are a finite resource and therefore will eventually run out. The reserve to production ratio of these fuels is shown in Figure 1. These values show how long reserves will last if consumption continues at the same rate. The global reserves of crude oil were estimated to be at 1732.4 billion barrels, meaning it would take 53.5 years until the exhaustion of supplies, assuming current consumption rates [1]. While new reserves of oil are continuing to be explored, the reliance on fossil fuels must be limited due to its adverse effects on the environment. Emissions of greenhouse gases (GHGs) such as carbon dioxide (CO_2_), methane (CH_4_), and nitrous oxide (N_2_O) have been linked with negatively altering the Earth’s climate. It has been well documented that the use of fossil fuels has resulted in global warming, with average surface temperatures reaching 1 °C above pre-industrial levels in 2017. This rise in global temperature can be correlated with the increased dependence on fossil fuels post-Industrial Revolution. From 1750 to 2011, the cumulative anthropogenic CO_2_ emissions totaled 2040 ± 310 GtCO_2_. Of the total increase in greenhouse gas emissions from the combustion of fossil fuels and industrial processes, 78% was directly from CO_2_ emissions. In 2018, carbon emissions grew by 2%, the highest rate in 7 years, with natural gas fueling energy growth [2]. With reserves of conventionally used fossil fuels depleting, increasing energy demand, and improved awareness toward global warming and climate change, significant importance has been placed on finding sustainable, environmentally friendly, and economically viable alternative sources of fuels and chemicals.

In recent years, wind and solar have emerged as viable sources of electrical energy. In 2018, power generation by renewable energy increased by 16%, with wind contributing 142 TWh and solar 131 TWh [2]. Overall, wind accounts for 50% of renewable generation, in comparison with the 24% provided by solar. While wind and solar can address the need for electrical energy demand, alternative renewable sources are required for transportation and heating fuels and sources of chemicals. Currently, over half of a barrel of crude oil is refined into transportation fuels. One barrel gives 10.04 gallons of diesel (22%) and 19.36 gallons of petrol (43%). In terms of chemical production, 4% of oil produced worldwide is used for chemical and plastic production [3]. Currently, the transport sector relies on petroleum, accounting for 96% of the transport energy. The use of biofuels could allow for a reduction in annual GHG emissions by ~52 million metric tons (MT) by 2030 (19% reduction) and by ~194 million MT by 2050 (47% reduction). The EU directive stipulated that, by 2020, 10% of transport energy must be derived from biofuels; however, according to the Department of Transport statistics, by 2023, the UK met only 3% of the renewable biofuels target.

**Figure 2:**
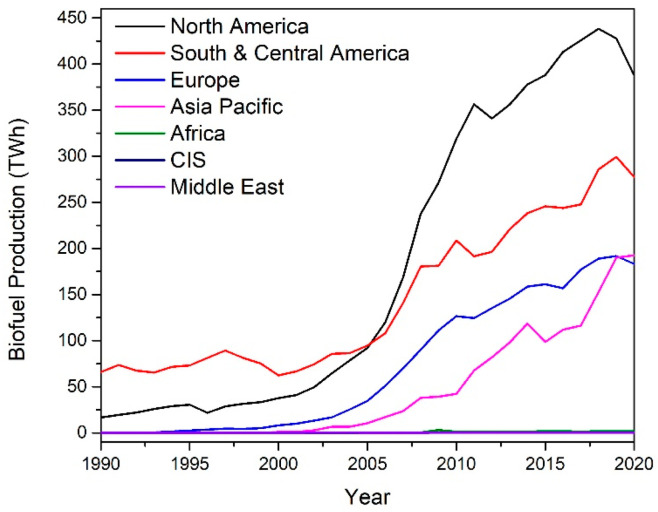
Biofuel production by region [2].

Biomass has emerged in recent years as a potential feedstock for the sustainable production of renewable fuels and chemicals. The biorefinery concept has come to the fore as a possible solution to this issue. The International Energy Association Bioenergy Task 42 has defined a biorefinery as “the sustainable processing of biomass into a spectrum of marketable products and energy”. The spectrum of marketable products and energy consists of intermediates and final products and includes food, feed, materials, chemicals, and energy (fuels, power, and/or heat). Typically, biorefineries can produce a form of biofuel product such as bioethanol or biodiesel. The growth in biofuel production since 1990 is shown in Figure 2. Biofuel production growth was above the 10-year average in 2018, with a 9.7% increase in production [2]. Bioethanol production totaled 60.4 mtoe (million tons oil equivalent), with North America being the largest producer at 56%. Biodiesel production totaled 34.9 mtoe in 2018, with Europe being the largest contributor at 37%. The combined bioethanol and biodiesel production is shown below in Figure 2.

As a fuel source, biodiesel has several advantages as it is renewable, non-toxic, and biodegradable. Upon combustion, biodiesel produces no sulfur, no net CO_2_, less carbon monoxide, zero particulate matters, no smoke, and no hydrocarbons [4]. Biodiesel is attractive as it can be used in diesel engines with little to no modifications or performance decline. Within the European Union, targets set by the Renewable Energy Directive (RED II) have increased the mandates of renewable transport fuels from 10% in 2020 to 14% in 2030. Similarly, in the United Kingdom, the Renewable Transport Fuel Obligation (RTFO), has set a target of a 12.4% biofuel blend by 2032. Hence, it is evident that biodiesel production will continue to increase in the next decade to meet these targets as countries push toward net-zero CO_2_. 

### 1.2. Glycerol: A Liability from Biodiesel Industry

**Figure 3:**
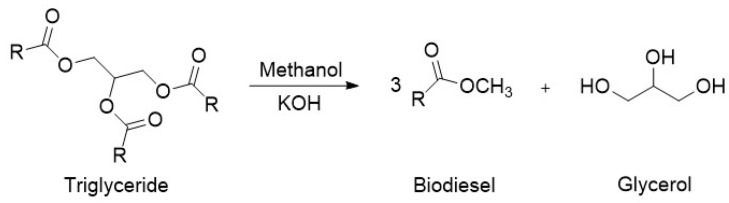
Reaction scheme for the production of biodiesel.

Typically, biodiesel is produced through the transesterification of triglycerides, contained in vegetable oils, with methanol to produce fatty acid methyl esters (FAMEs), as shown below in Figure 3 [5]. The reaction is catalyzed by alkalis such as sodium or potassium hydroxide. Due to its reversible nature, the reaction is normally performed with an excess of alcohol to ensure complete conversion of the vegetable oil.

A major problem associated with the production of biodiesel is the formation of a by-product glycerol, which accounts for 10 wt% of all biodiesel production. Glycerol or glycerine (IUPAC propane-1,2,3-triol, CAS:56-81-5) is a simple polyol with a molecular formula of C_3_H_8_O_3_. It consists of a propane molecule substituted with three hydroxyl groups at positions 1, 2, and 3. The structure of glycerol is shown in Figure 3. In its pure form, glycerol is colorless, non-toxic, odorless, and viscous. The properties of glycerol are shown in Table 1.

**Figure 4:**
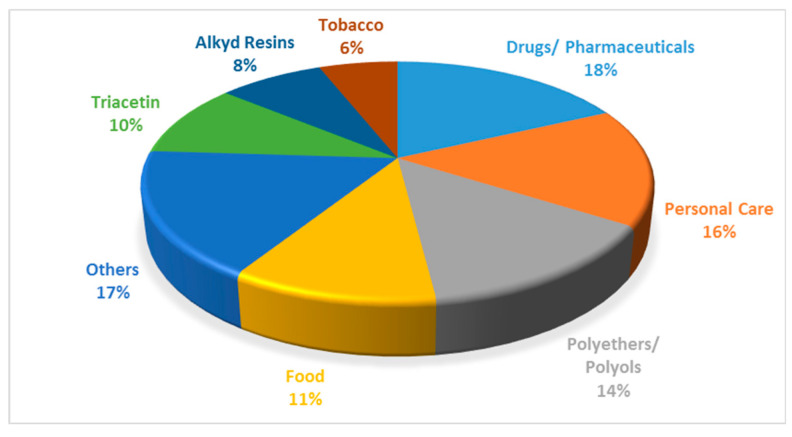
Various applications of glycerol. Reprinted/adapted with permission from Ref. [9]. 2007, John Wiley and Sons.

Pure glycerol has a wide range of uses, including the manufacture of drugs, cosmetics, toothpastes, urethane foams, synthetic resins, and ester gums [8]. It is also used as a miscellaneous or general-purpose food additive due to its non-toxic nature. The various applications of glycerol are shown in Figure 4.

The problem associated with the increase in biodiesel production is two-fold. Firstly, the increase in biodiesel production over the last 20 years has naturally led to an increase in the amount of by-product formation. The surplus of glycerol resulting from this increase has led to a market where the supply of glycerol is independent of the demand, resulting in a marked decrease in the price of glycerol [10]. In 1999, the oleochemical industry supplied 47% of the world’s glycerol, changing dramatically from 2009, where 64% of glycerol was supplied by the biodiesel industry. In 2014, the price of 80% crude glycerol was USD 0.24 kg^−1^, and the United State Pharmacopeia grade was USD 0.9 kg^−1^ [11]. This supply is expected to keep increasing in the next number of years [12]. One positive aspect of the decrease in the glycerol price is that it makes it an attractive feedstock to create value-added products; for instance, some of the traditional applications are shown in Figure 4. 

### 1.3. Upgrading Glycerol to Value-Added Glycerol Esters: The Key to Convert a Liability into an Asset

**Figure 5:**
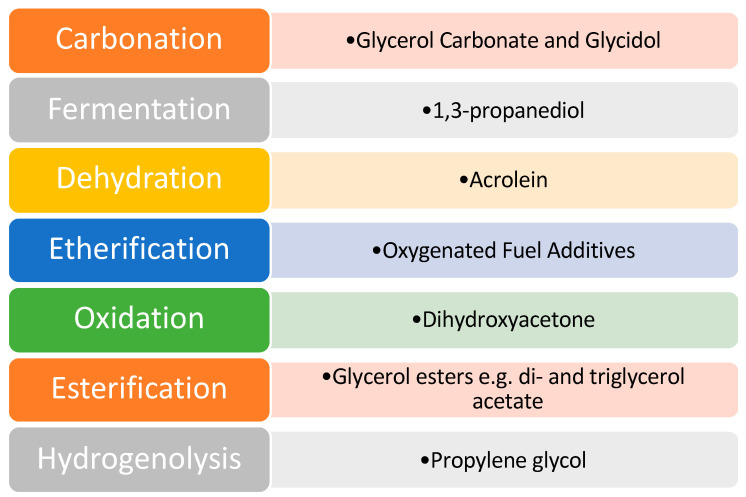
Pathways of glycerol valorization. Reprinted/adapted with permission from Ref. [9]. 2007, John Wiley and Sons.

Despite the issues associated with glycerol, it was identified as one of the top twelve platform chemicals by the United States Department of Energy. As a result of this, a great deal of scientific research has been directed toward this area to develop effective catalysts and efficient pathways of value addition. Various pathways that have been explored for glycerol valorization are shown in Figure 5.

The purity of glycerol produced from the biodiesel industry as a by-product is quite low and therefore unsuitable for most traditional applications. While the composition of crude glycerol varies from producer to producer, most of the crude glycerol includes impurities such as methanol, water, soap, and FAMEs [13]. Crude glycerol will also contain smaller amounts of glycerides, unreacted free fatty acids, and ash. Also, depending on the efficacy of post-treatment in the plants, residual alkali, such as NaOH or KOH, can remain in the crude glycerol resulting in a high pH level. The characterization of crude glycerol is important as it can affect which applications it is appropriate for. Often, the large cost associated with refining crude glycerol can only be afforded by large-scale manufacturers, and it is not an economically viable option for small- or medium-scale manufacturers. It is therefore important to find ways of adding value to this waste product, not only to promote a circular economy and improve sustainability, but also to improve the economic viability of the biorefinery industry [14]. In this context, the esterification of glycerol is among the most-employed organic transformations for upgrading glycerol to glycerol esters. The key advantage here is the application of glycerol esters as a high-energy-density “drop-in” fuel additive, which can be blended back into the biodiesel pool. Thus, this accomplishes the conversion of the liability from the biodiesel industry into assets while adhering to the principles of the circular economy approach and improving the process economics and profitability of the overall biorefinery concept. In continuation of our group’s interest in a biomass-derived drop in fuels [15,16,17,18,19,20,21,22,23,24] and an environmental catalysis [25,26,27,28,29,30,31,32,33,34,35,36,37,38], herein, we have critically reviewed the upgrading of glycerol to glycerol esters as the products. 

The esterification of glycerol with acetic acid produces monoacetin (MA), diacetin (DA), and triacetin (TA) acetyl esters, which have added economic value compared to the crude glycerol waste from the biorefinery. Triacetin has been shown to be an effective fuel additive when blended with biodiesel leading to reduced CO_2_ emissions, hydrocarbons, and particulate matter [39], while also leading to a reduction in cloud point and pour point [40]. The effective production of triacetin could be a two-edged sword in combatting the issues of biodiesel production while simultaneously dealing with the surplus of glycerol and also providing an additive that can be blended with biodiesel to improve fuel properties. The triacetin market has been forecasted to grow from USD 255.6 million to USD 362.1 million by 2026 [41]. The various uses of these esters are summarized in Table 2. The reaction can also be performed using acetic anhydride as an acetylating agent; however, safety issues can arise due to the formation of explosive vapor/air mixtures [11]. Acetic acid is also cheaper when compared to acetic anhydride, at USD 0.5 kg^−1^ and USD 0.98 kg^−1^, respectively [42].

**Figure 6:**
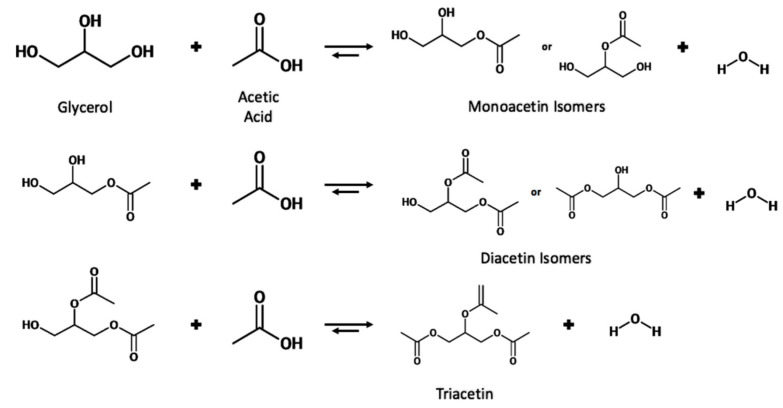
Reaction scheme for the esterification of glycerol with acetic acid.

The reaction proceeds stepwise with the substitution of an acetyl group with the hydrogen of a hydroxyl group to form the ester and water. Due to the three hydroxyl groups present in glycerol, the substitution can occur for each group, producing a water molecule each time. The reaction scheme is shown in Figure 6.

Due to the reversible nature of the reaction, various techniques have been utilized to shift the equilibrium toward the right-hand side. These techniques include increasing the temperature of the reaction, removal of in-situ water, and increasing the molar ratio of glycerol to acetic acid.

### 1.4. Reaction Mechanism for Esterification of Glycerol

The reaction can occur through a Brønsted acid- or Lewis acid-catalyzed mechanism, although both mechanisms are similar in nature.

**Figure 7:**
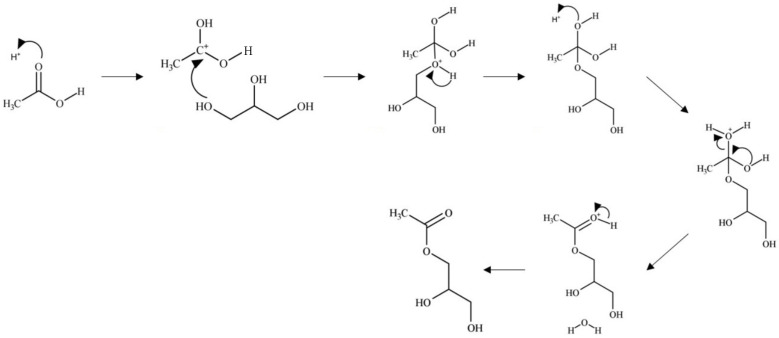
Brønsted acid-catalyzed glycerol esterification with acetic acid.

In the Brønsted acid mechanism, as shown in Figure 7, the protonation of the acetic acid carbonyl occurs via a proton from the catalyst [46]. The resulting carbocation formed undergoes a nucleophilic attack from an oxygen of a glycerol hydroxyl leading to the loss of a proton. An ester bond is formed through the hydroxyl groups of acetic acid, which undergo fast equilibrium proton exchanges resulting in the elimination of water. The catalyst is regenerated through the elimination of the excess proton. This mechanism occurs similarly with the remaining hydroxyl groups of glycerol. 

**Figure 8:**
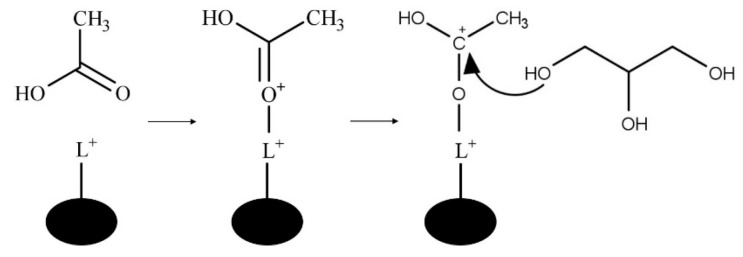
Lewis acid-catalyzed glycerol esterification with acetic acid where L^+^ is a Lewis acid site.

The Lewis acid-catalyzed mechanism is shown in Figure 8. In the Lewis acid-catalyzed mechanism, a metal cation acts as an electrophile to form the carbocation via acetic acid carbonyl oxygen and the Lewis acid site of the catalyst [11].

## 2. Nanostructured Solid/Liquid Acid Catalysts for Glycerol Esterification

### 2.1. Homogeneous Catalysts Used in the Esterification of Glycerol

Mineral acid catalysts, such as H_2_SO_4_ and HCl, have typically been used in esterification reactions. These catalysts are associated with several drawbacks. They are hazardous to handle, corrosive, and lead to large volumes of process waste due to the need for quenching and separating acids, and the acids are normally destroyed in quenching and neutralization, so they are non-reusable [47]. With increased importance placed upon green chemistry practices, the need to develop catalysts which can overcome these drawbacks is high.

Ionic liquids (Ils) have emerged as potential replacements of mineral acid catalysts as they have several benefits such as good thermal stability, ease of handling, and, importantly, good recyclability. The structure and performance of various Ils are reported in Table 3. As Ils are composed of a cation and an anion, the chemical and physical properties of Ils can be changed by adjusting the composition of the ions to produce functionalized ionic liquids. One such form of functionalized ionic liquids is the Brønsted acidic ionic liquids (BAILs), which gain functionality through covalently bonded sulfonic acid species (-SO_3_H) or Brønsted acidic counter anions (HSO_4_^−^, H_2_PO_4_^−^) [48].

The use of ionic liquids for the esterification of glycerol with acetic acid was first reported by Deng et al. [49]. Using a combination of aluminum (III) chloride and 1-butylpyridinium chloride, the conversion of glycerol and the selectivity of the products were found to be comparable to sulfuric acid.

Li et al. first reported the use of SO_3_H-functionalized ionic liquids, which were composed of [HSO_3_^−^pmim] as the cation and a range of counter anions such as [HSO_4_]^−^, [PTSA]^−^, [H_2_PO_4_]^−^, [BF_4_]^−^, and Cl^−^ [50]. The use of [PTSA] provided the most active catalyst, and [HSO_4_] also provided excellent activity while remaining active upon recycling. The effect of double SO_3_H-functionalized ionic liquids was investigated by Liu et al. [51]. The double SO_3_H-functionalised ionic liquids outperformed those with only one SO_3_H group, due to a higher level of Brønsted acidity. Similarly, [HSO_4_]^−^ was found to be the most active counter anion against [NTf_2_]^−^ and [tos]^−^. Huang et al. reported the use of heteropolyacid-based ionic liquids consisting of pyridinium propyl sulfonate, tungstophosphoric acid, and acetic acid achieving an 85.9% selectivity to triacetin after 4 h at 105 °C with continuous water removal [45].

Keogh et al. reported the use of a range of nitrogen-based Brønsted acidic ionic liquids based on alkyl pyrrolidone and alkyl amine cations [52]. Among all ionic liquids studied, N-methyl-2-pyrrolidinium hydrogen sulfate [H-NMP][HSO_4_] was found to be the most active catalyst. The effect of significant reaction parameters on selectivity to the tri-substituted product, triacetin, was modeled using a design of experiment (DoE) approach with a response surface methodology involving a central composite design. Among the reaction parameters evaluated, temperature had the highest influence on product selectivity, followed by the glycerol to acetic acid molar ratio, and the model also showed dependence on the synergistic interaction between the temperature and mole ratios. 

In a separate study, Liu et al. showed the synergistic effect of both Brønsted and Lewis sites where a Brønsted–Lewis acidic ionic liquid outperformed solely Brønsted acidic and solely Lewis acidic ionic liquids [53]. Sun et al. prepared rod-like carbon-based ionic liquids, which were functionalized with sulfonic acid [44]. The prepared ionic liquids were evaluated in their ability to produce triacetin from glycerol. The [PrSO_3_HN][SO_3_CF_3_]/C-2 ionic liquid was the most active, giving a high yield of triacetin of 74.8% after 8 h. Podolean et al. prepared manganese oxide modified with ionic liquids via a thin layer of [Bmim][NTf_2_] or [Bmpyr][NTf_2_] [54].

Heteropolyacids are a class of strong Brønsted acids consisting of (i) metal, e.g., tungsten, molybdenum, or vanadium, (ii) oxygen, (iii) p-block element, e.g., silicon, phosphorus, or arsenic, and (iv) acidic hydrogen atoms [47]. Typically, tungstophosphoric acid (TPA), silicotungstic acid (STA), and phosphomolybdic acid (PMA) are used. While heteropolyacids tend to be highly soluble in the reaction mixture, they can be reused in a series of recycling steps. Gonçalves et al. reported the use of TPA, which offered comparable activity to that of sulfuric acid and p-toluenesulfonic acid [55]. The protons of heteropolyacids can be exchanged with metal ions to improve the activity and thermal stability and also tune the solubility of the heteropolyacid in the reaction media. Although an exchange with metal ions can often lead to insoluble heteropolyacid salts, it can also have a limited effect on the solubility. Da Silva et al. investigated the effect of exchanging the protons of TPA, PMA, and STA with Lewis acidic metals such as Cu, Co, Mn, and Fe [56]. STA was found to be the most active heteropolyacid, followed by TPA, and finally PMA. Iron was found to be the most active metal regardless of the heteropolyacid. A complete exchange of the protons of STA with iron resulted in a partial soluble catalyst with increased activity. When exchanging with Sn, Chaves et al. found TPA to be more active than STA and PMA [57].

Soluble tin (II) chloride has also been reported for the reaction [55]. The less corrosive Lewis acid was found to have comparable activity to sulfuric acid and produced less reaction by-products.

**Table 3:** Structure and performance of various ionic liquids in the esterification of glycerol with acetic acid.

Structure	Reaction Conditions	Performance	Ref.
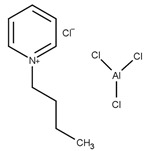	T = 75 °C t = 2 h Molar ratio acetic acid:glycerol = 3:1 Catalyst = 1 mL	*C* = 100% *S* = 17.1% MA 58.8% DA 24.1% TA	[49]
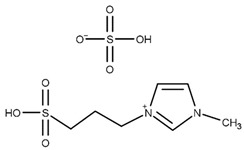	T = 120 °C t = 6 h Molar ratio acetic acid:glycerol = 8:1 Catalyst = 6.25 mol%	*Y* = 95.6% TA	[50]
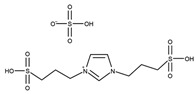	T = 100 °C t = 30 min Molar ratio acetic acid:glycerol = 8:1 Catalyst = 0.1 mol%	*C* = 95.0% *S* = 43.1% MA 51.4% DA 5.5% TA	[51]
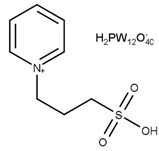	T = 105 °C t = 6 h Molar ratio acetic acid:glycerol = 10:1 Catalyst = 2.5 mol%	*C* = 100% *S* = 2.3% MA 40.0% DA 57.7% TA	[45]
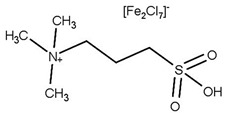	T = Reflux t = 4 h Molar ratio acetic acid:glycerol = 5:1 Catalyst = 0.3 mol% Solvent = 30 mL toluene	*Y* = 98.6% TA	[53]

### 2.2. Heterogeneous Catalysts Used in the Esterification of Glycerol

Solid acid catalysts can offer easier separation when compared to reusable homogeneous catalysts. Often, a simple filtration can separate the catalyst from the reaction mixture. Solid acids also give generally clean and selective reactions with high purity products [47]. The characteristics of solid acid catalysts, such as acidity, catalyst texture, and surface morphology, can be tuned to offer a high conversion of glycerol and a high selectivity of DA and TA products [11].

#### 2.2.1. Metal Oxide Catalysts

Hu et al. investigated the abilities of a wide range of metal oxides to catalyze the esterification on glycerol with acetic acid, with a high selectivity to diacetin [58]. When compared to a blank experiment, which gave a 45.2% conversion of glycerol and a selectivity to diacetin of 12.6%, only marginal improvements were observed with Sb_2_O_3_, Bi_2_O_3_, SnO_2_, TiO_2_, and Sb_2_O_5_. A higher glycerol conversion of 94.5% and a selectivity to diacetin of 46.8% were observed using antimony pentoxide (Sb_2_O_5_). Good selectivity to diacetin is often hard to achieve, as low activity catalysts will mainly show a high selectivity to monoacetin, with high activity catalysts showing a high selectivity to triacetin. The Sb_2_O_5_ catalyst also showed good reusability with no change in conversion or selectivity after six runs.

**Figure 9:**
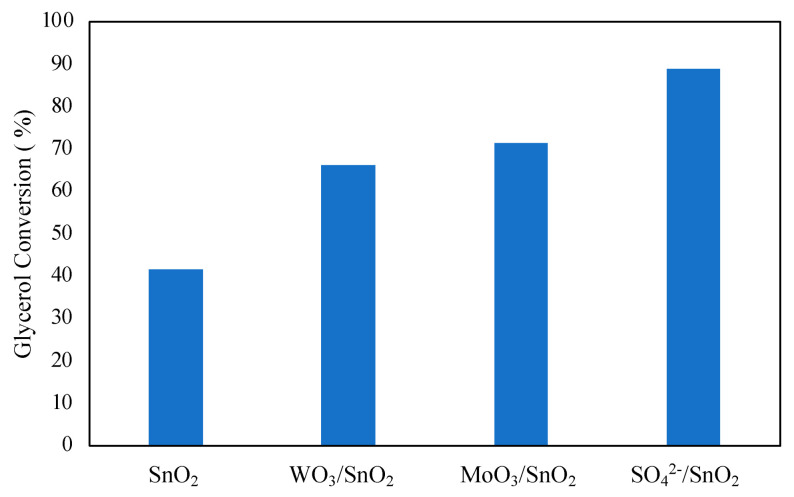
Effect of promoters of SnO_2_ on glycerol conversion. Reprinted/adapted with permission from Ref. [59]. 2014, American Chemical Society.

The effect of adding promoting species to metal oxides was investigated by Mallesham et al. [59]. Promoting species SO_4_^2−^, MoO_3_, and WO_3_ were added to tin oxide using a wet impregnation method. The addition of promoting species was found to improve glycerol conversion, as shown in Figure 9. The highest glycerol conversion was observed with the SO_4_^2−^-promoted tin chloride at 89%. The enhanced performance of the catalyst can be explained by the high number of acidic sites at 186.98 μmol/g with an abundance of superacidic sites. Reusability of the catalyst was found to be quite low, with glycerol conversion decreasing to 51% on the 4th cycle, also leading to decreased selectivity to diacetin and triacetin with each cycle. The quick decline in catalytic activity was attributed to decreased acidic sites and BET surface area of the catalyst after each cycle. It can then be noted from the results of Mallesham et al. that a high number of acidic sites on the catalyst is imperative for the high conversion of glycerol in the reaction.

Reaction conditions: 70 °C, 120 min, molar ratio of acetic acid to glycerol of 1:1, and 5 wt% catalyst.

Sulfate ions (SO_4_^2−^) have also been used by Reddy et al. to improve the activity of a ceria and zirconia mixed oxide species [60]. The sulfonated mixed oxide showed an increased surface area (49 to 92 m^2^/g) and increased strength and number of acid sites from the pristine mixed oxide. Sulfonated ceria–zirconia has also been reported by Kulkarni et al. [61]. Sulfate ions have been shown to improve the activity of titania and silica mixed oxides [62]. The mixture of these two oxides, consisting of 13.8 wt% TiO_2_, generated a higher number and strength of acid sites. 

Similarly, Reddy et al. investigated the effect of promoting species (TiO_2_, WO_x_, and MoO_x_) on zirconium oxide [63]. Of the catalysts prepared, a nearly 100% conversion after 3 h at 120 °C was observed with WO_x_/TiO_2_-ZrO_2_ and MoO_x_/TiO_2_-ZrO_2_. A high selectivity toward diacetin was also observed with 40.01% and 40.45%, respectively. 

#### 2.2.2. Ion-Exchange Resins

One class of solid acid catalysts are ion-exchange resins, which exchange ions between themselves and the reaction media [47]. The resins are usually copolymers of divinylbenzene or styrene and ion-exchanging functional groups. Dosuna-Rodriguez et al. evaluated the ability of several ion exchange resins to catalyze the reaction [64]. Unlike other reports in the literature on this topic, reactions were carried out with an excess of glycerol. As glycerol is a low-value molecule, this keeps the costs of reactions low, but it also results in the desired shift of equilibrium to the right to promote the formation of the products. The results of the various ion-exchange resins are shown in Table 4. Amberlyst-36 was tested to determine the reusability of the catalyst, and no significant change in activity was observed after four cycles. 

Zhou et al. showed that Amberlyst-15 could obtain high conversions and selectivity to DA and TA when combined with an excess of acetic acid [65]. Amberlyst-15 and -70 were investigated by Kale et al. with the use of toluene as an entrainer aiming for a high selectivity to triacetin [66]. The use of toluene as an entrainer was found to be key for the conversion and product selectivity. Without toluene, A-70 gave only 9.3% selectivity to TAG, increasing markedly to 45.8% with the use of toluene. A maximum TAG selectivity of 95.3% was observed after 24 h using A-70.

A polysulfone catalyst which was 2 times as acidic as Amberlyst-15 was developed by Wang et al. [67]. The catalyst was more active due to its increased acidity and swelling properties. Moreover, the catalyst showed good stability upon reuse, while Amberlyst-15 underwent deactivation upon reuse. This was attributed to the unstable bonding of acid groups via the post-sulfonation method. 

Other commercial ion-exchange resins such as Dowex Monosphere 650C (Dow Chemical Company, Midland, MI, USA) and Purolite CT-275 (Purolite Corporation, Llantrisant, UK) have also been reported [68,69,70].

#### 2.2.3. Zeolite-Based Solid Acids

Zeolite-based solid acids are aluminum silicates which form a regular crystal lattice, catalyzing reactions in their internal cavities [47]. Gonçalves et al. compared the zeolites HUSY and HZSM-5 against the acidic catalysts of Amberlyst-15, K-10 montmorillonite, and niobic acid [71]. The results showed poor activity of the zeolite catalysts with HZSM-5 and HUSY giving a 30% and 14% glycerol conversion, respectively, after 30 min. For both, monoacetin was the major component with small amounts of diacetin; however, no triacetin was detected for either catalyst. The low conversion was attributed to diffusion problems of the esters within the catalyst pores and deactivation of acid sites on the catalyst.

The effects of Zr modification on mordenite (M) and hierarchical mordenite (M1) for catalyzing the reaction was investigated by Popova et al. [72]. H-mordenite was prepared with acidic treatment of the parent mordenite, with Zr added by incipient wetness impregnation. The highest conversion of glycerol observed after 3 h at 100 °C was with Zr/M1 at 93.5%, with an impressive yield of triacetin of 69.2%. Under the same conditions, it was observed that the acidic treatment of mordenite gave higher activity than the parent mordenite, which can be attributed to the increase in pore size from 0.9 nm to 1.6 nm. Higher conversions of glycerol to valuable triacetin were also observed for the M1 catalysts. The Zr-modified catalysts exhibited increases in glycerol conversion from their parent catalysts, which can be attributed to the increased number of Brønsted and Lewis acid sites. The results from this experiment are summarized in Table 5.

Gao et al. compared the activity of a graphene oxide catalyst against the zeolites ZSM-48, ZSM-5, and H-mordenite [73]. After 1 h at 120 °C with a glycerol to acetic acid ratio of 1:10, the catalyst produced an average conversion of glycerol, with the activity following the trend ZSM-5 > H-mordenite > ZSM-48. Despite an average conversion of glycerol, these catalysts produced high yields of diacetin, with ZSM-5 giving a diacetin yield of 62.2%. The activity of ZSM-5 could be improved through the incorporation of 5 wt% cerium in the structure, with glycerol conversion increasing from 76.43 to 98.32% under the same conditions. However, only monoacetin and diacetin were reported.

#### 2.2.4. Silica-Based Solid Acids

**Figure 10:**
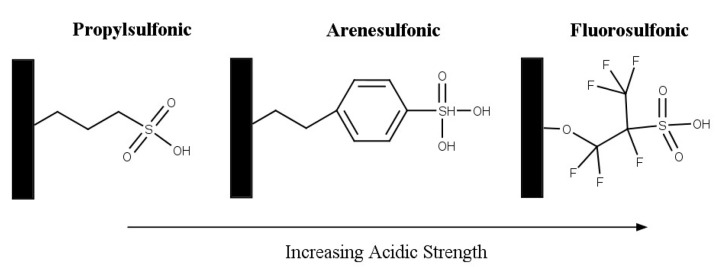
Structure of sulfonic acid groups on mesoporous silica. Reprinted/adapted with permission from Ref. [75]. 2007, American Chemical Society.

Silica-based solid acids are also widely investigated as a support in catalysis as they are easily available and inexpensive [47]. The addition of sulfonic groups to mesoporous materials, commonly MCM-41, HMS, and SBA-15, produce solid acid catalysts. The acid catalysts have the following properties: high surface area (≥1000 m^2^/g), large pore sizes (2 nm–20 nm), and relatively high acid strengths [74,75,76]. Melero et al. investigated acidic mesoporous silica for the acetylation of glycerol [75]. Three materials were prepared by incorporating phenyl, propyl, and fluorosulfonic groups with SBA-15 material. The structure of the sulfonic acid groups is shown in Figure 10. The incorporation of more electron-withdrawing groups, such as phenyl and fluoro, results in an increased acid strength. The activity that was observed followed the same trend of the sulfonic group’s acid strength: fluorosulfonic > arenesulfonic > propylsulfonic. After 2 h, the highest selectivity to di-and triacetin was shown by Ar-SBA-15; however, it was noted that after 4 h, most of the materials achieved around 80% selectivity to di- and triacetin.

The effect of niobium on the formation and stability of sulphonic species in these materials was examined by Trejda et al. who prepared silicate- and niobiosilicate SBA-15-type catalysts modified with MPTMS (3-mercaptropropyl)trimethoxysilane [77]. It was found that the incorporation of niobium into SBA-15 improves the oxidation of –SH by hydrogen peroxide to sulphonic species; however, it did not increase the stability of the species. A maximum conversion of 94% was found with MP-Nb-SBA-15-32 after 4 h, with a selectivity to di- and triacetin of 52% and 37%, respectively. 

Khayoon et al. investigated the promotional effect of yttrium on the activity of SBA-3 [78]. The incorporation of yttrium was found to increase the surface area of SBA-3 from 1462 m^2^/g to 1568 m^2^/g. The increased activity of the 3 wt% Y/SBA-3 catalyst was attributed to the combination of a higher surface area and increased stability of the crystalline SBA-4 material after yttrium grafting. 

Other silica-based materials include mesostructured cellular foams (MCFs) which have walls formed from silica [79]. MCFs are uniform spherical cells with large surface areas up to ca 900 m^2^g^−1^ interconnected by uniform windows (7–20 nm) forming a continuous porous system. Stawicka et al. [79] synthesized niobium- and tantalum-containing MCFs modified with MPTMS. The highest conversion of glycerol was achieved with MP-TaMCF, but MP-NbMCF gave the highest yield of triacetin (38% after 4 h at 398 K). The choice of metal was found to not affect the amount of MPTMS anchored and instead affected the number of Brønsted acid sites. While MP-TaMCF had the highest number of Brønsted acid sites, MP-NbMCF was found to have the strongest Brønsted acid sites. Stawicka and co-workers found that the strength of the Brønsted acid sites was the most important factor in determining the yield of the valuable product triacetin.

#### 2.2.5. Heteropolyacids (HPAs)

To combat the disadvantages associated with the solubility of heteropolyacids in the reaction mass, heteropolyacids can be supported on an appropriate carrier. Similarly, exchanging the protons of the heteropolyacids with a metal ion can result in a heteropolyacid salt, which is insoluble in the reaction mass. 

Metal oxides have been used as supports for heteropolyacids. Zhu et al. investigated glycerol esterification using three zirconia-supported HPAs: TPA, STA, and PMA [80]. Previously Zhu et al. reported that ZrO_2_-supported STA was the most active and had the highest stability when compared to supports such as y-Al_2_O_3_, activated carbon, TiO_2_, and SiO_2_ [81]. From the results, it was shown that the acid strength of the HPAs followed the trend of TPA > STA > TMA. STA had the highest Brønsted acidity at 92.2 μmol/gcat with the results of the glycerol esterification reflecting this. When the STA/ZrO_2_ conversion of glycerol reached 96.4% after only 1 h at 120 °C, the selectivity was 60.5% and 11.2% for di- and triacetin, respectively. When the conversion increased to 100% after 4 h, the selectivity increased to 61.3% and 32.3%, respectively. The catalyst also exhibited good reusability after four runs with negligible change in the conversion, whereas TPA and PMA exhibited a decreased conversion.

Jagadeeswaraiah et al. doped zirconia with cesium and used it as a support for TPA [82]. The loading of TPA onto the cesium-doped zirconia resulted in a partial exchange of the TPA protons with the cesium ions. The presence of cesium was found to increase the activity of the catalyst as a result of the increase in strength and the number of acid sites. A full exchange (TPA/Cs_3_-ZrO_2_) was the least active exchanged catalyst due to the absence of residual protons. The optimal catalyst was found to be TPA/Cs_2_-ZrO_2_, which has two protons exchanged with two cesium ions. TPA has also been supported on niobium pentoxide [83].

Silica-based materials have been widely used as supports for the incorporation of HPAs. TPA_3_/MCM-41 gave the highest yield of 87% after 6 h at 100 °C, with a selectivity to di- and triacetin of 60% and 15%, respectively. Ferreira et al. prepared TPA on a silica matrix prepared by the sol–gel and wet impregnation methods [84]. Catalysts prepared by sol–gel were more active than those prepared by wet impregnation. The loading of TPA by the sol–gel method resulted in an increase in the SA from 223 to 254 m^2^/g, with the presence of very strong acid sites. SBA-15 was found to be an effective support for PMA [85]. A 15 wt% loading of PMA/SBA-15 gave complete glycerol conversion after 1 h and a combined DA and TA selectivity of 86% after 3 h.

Magar et al. investigated the activity of different HPAs using polyvinylpyrrolidone as a support [86]. The activity of the HPAs was found to be TPA > STA > PMA, with the activity corresponding well with the acidic strength of the catalysts. Zeolites such as USY and activated carbon have also been used as supports [87,88].

Zhu et al. synthesized Ag-exchanged TPA (or HPW) catalysts using an ion-exchange method [89]. The trend for the catalyst activity from the highest to lowest was Ag_1_PW > Ag_2_PW > Ag_3_PW. Glycerol conversion with Ag_1_PW reached 100% within only 45 min. The conversions after 15 min at 120 °C with 1 wt% catalyst and a glycerol to acetic acid mole ratio of 1:10 are shown in Table 6. The Ag1PW showed similar activity after five cycles, exhibiting good reusability.

Similarly, TPA can be exchanged with cesium to produce an insoluble cesium phosphotungstate salt [90]. The CsTPA catalyst outperformed H-beta, K-10, and sulphated zirconia due to a high number of acid sites (1.87 mmol/g). The catalyst also out exhibited a higher selectivity to triacetin than Amberlyst-15. Sun et al. reported the use of an indium-exchanged TPA catalyst [91]. The catalyst was found to exist in a nanotube-like structure, which combined with the presence of Lewis and Brønsted acid sites led to the selectivity formation of MA.

Keogh et al. investigated the kinetics of the esterification of glycerol with acetic acid using partial tin-exchanged TPA supported on montmorillonite K-10 as catalysts [18]. Partially exchanging the H^+^ ion of TPA with Sn (x = 1) increased the acidity of the catalyst and showed an increase in the catalytic activity as compared to the supported TPA/K-10 catalyst. Among various catalysts, Sn_1_-TPA/K-10 proved to be the most active catalyst for glycerol esterification. The Langmuir–Hinshelwood (L–H) dual-site model was able to describe the experimental data with high agreement between the experimental and calculated results. The tin-exchanged TPA supported on montmorillonite K-10 catalysts were found to be robust and shown to recycle four times without loss of activity. 

#### 2.2.6. Carbon-Based Catalysts 

In 2015, Gao et al. reported the esterification of glycerol and acetic acid using a graphene oxide catalyst [73]. Under reaction conditions of 120 °C, 1:10 molar ratio of glycerol to acetic acid, and 0.1 g catalyst, glycerol conversion reached 98.5% after 1 h, with a selectivity to di- and triacetin of 60% and 24.5%. The high catalytic activity of graphene oxide for this reaction can be directly attributed to the high number of –SO_3_H groups on the catalyst surface, which was measured to be 0.378 mmol/g. The catalyst also showed good reusability with no decline in conversion or variation in the distribution of products. 

Sanchez et al. prepared porous carbon-based catalysts by the sulfonation of carbonized sucrose [92]. Direct synthesis carbonization (DC) and template-assisted carbonization (TAC) were used followed by the functionalization of the carbon with the –SO_3_H groups. TAC-673 was observed to have the highest density of the sulfonate groups at 1.35 mmol/g. In the esterification of glycerol with acetic acid (1:9), in the reaction temperature range of 378 to 473 K, all reactions using the DC and TAC gave conversions of higher than 99.6%, with a significant increase in the selectivity to triacetin from 17% (at 378 K) to 50% (at 473 K). Willow catkins, a low-cost biomass, has also undergone carbonization to produce a catalyst [93]. The sulfonation of activated carbon was also reported by Khayoon et al. [94].

Okoye et al. had a novel solution for the excess of crude glycerol, using it in the synthesis of an acid catalyst involving sulfonation and carbonization, which could then catalyze the acetylation of glycerol [95]. The carbon catalyst is irregularly shaped with few pores, and it contains both Brønsted acidic sulfonate groups and Lewis acidic carboxylic groups. After seven recycles, the catalyst showed constant acid density, indicating good reusability of the catalyst. 

Carbon spheres and xerogels can be modified with sulfonic acid groups to produce active acidic catalysts [96]. Both decreased in surface area upon sulfonation, but it was most dramatic with carbon spheres, decreasing from 371 to 11 m^2^/g. Sulfonated xerogel had an acidity of 1.19 mmol/g, and carbon spheres had an acidity of 2.77 mmol/g. As a result, sulfonated carbon spheres were more active, providing a similar level of activity to Amberlyst-15. 

#### 2.2.7. Others 

Troncea et al. reported the use of hydroxylated magnesium fluoride catalysts [46]. The mesoporous catalyst contained a mixture of Lewis and Brønsted acid sites. A higher Lewis to Brønsted acid site ratio was found to favor the formation of DA and TA due to the two-fold effect of the Lewis acid sites acting as a catalyst and dehydrating site. Tangestanifard et al. investigated the use of bentonite which was functionalized with arenesulfonic acid [97]. The modified clay exhibited a marked decrease in the SA and pore volume but an increase in the number of acidic sites (1.7 mmol/g). Such an increase in conversion was also found when compared to H-bentonite from a 67% conversion to 100%. Utilizing toluene as an entrainer, complete conversion could be achieved, with a selectivity of 26% DA and 74% TA. The functionalization of phenolic resins and polyphenylene sulfide fabrics with SO_3_H groups have been reported [98,99].

#### 2.2.8. Comparison of Homogeneous and Heterogeneous Catalysts

The performance and reaction conditions of various homogeneous and heterogeneous acid catalysts in the esterification of glycerol with acetic acid is shown below in Table 7. It can be noted that homogeneous catalysts tend to outperform heterogeneous catalysts in this reaction at relatively lower catalytic loading. Ionic liquid catalysts can provide the benefits of homogeneous catalysis while also being reusable and recyclable. The ionic liquid [HSO_3_-pmim][HSO_4_] was among the most active yielding at 95.6% TA (reaction conditions: 120 °C, 8:1 acetic acid to glycerol mole ratio, 6.25 mol% catalyst loading, and 360 min). This has shown that ionic liquids can be highly efficient catalysts for this reaction. However, there are disadvantages to ionic liquids such as [HSO_3_-pmim][HSO_4_]. The use of expensive components and multistep synthesis methods limit the industrial use of these catalysts. To overcome these disadvantages, the development of more cost-effective acidic ionic liquid catalysts for the reaction should be pursued further. Cost-effective and easily synthesized ionic liquids such as those based upon alkyl pyrrolidone and alkylamine cations with a hydrogen sulphate anion have not yet been explored in the research. 

Similarly, heteropolyacids have been shown to be effective catalysts for the reaction. Tin-exchanged tungstophosphoric acid (Sn_1.5_PW_12_O_40_) gave a 96% conversion and a 40% selectivity to TA after 180 min (reaction conditions: 70 °C, 12:1 acetic acid to glycerol mole ratio, and 0.78 mol% catalyst loading) [18]. The disadvantages of this catalytic system occur from difficulties in recycling of the catalyst after the reaction. The use of a support has shown to be effective in catalyst heterogenization. Further investigation should focus on the use of more acidic catalyst supports such as K-10 montmorillonite clay. The effect of cost-effective metal ion substitution, such as tin, should also be considered to tailor the strength of the acid site.

#### 2.2.9. Techno-Economic Assessment and Sensitivity Analysis of Glycerol Esterification

**Figure 11:**
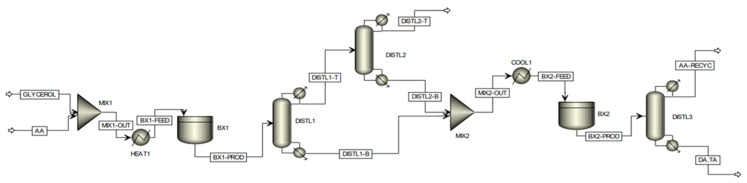
Proposed batch modeling flowsheet of two-stage glycerol esterification process on ASPEN Plus. Reprinted/adapted with permission from Ref. [24]. 2023, American Chemical Society.

Recently, Keogh et al. from our group investigated the economic feasibility of the production of DA and TA via a two-stage process using Aspen Plus^®^ (https://www.aspentech.com/) [24]. The assessment of the commercial viability of the partial tin-exchanged TPA supported on a montmorillonite K-10 catalyst at scale was conducted by a detailed techno-economic analysis, considering a plant with a fixed annual capacity for processing 100,000 tons of crude glycerol. The proposed batch modeling flowsheet of the process is shown in Figure 11. Based on the experimental data, it was not feasible to achieve complete selectivity to di- and triacetin by using a single batch reactor stage, hence a two-stage reaction process was considered. Following the first batch reactor, the product enters a distillation column, defined as ‘DISTL1′. The purpose of this preliminary column is to remove all water co-generated by the esterification reactions and thus remove the inhibiting presence of water from the reaction medium, which restricts the position of equilibrium. Due to the proximity in boiling points of acetic acid and water, a secondary column, ‘DISTL2′, is required to effectively recover the acetic acid lost in the distillate of the primary column; such acetic acid is recovered efficiently in this column, leaving with high purity within the bottoms stream where it is subsequently utilized in the second stage reaction. The distillate of the secondary distillation column consists of an essentially pure water stream, with only trace quantities of acetic acid, which can subsequently be disposed of safely, posing no threat to the environment. Due to the high acetic acid demand required to assist in driving the position of equilibrium toward the formation of the desired higher esters, an effective acetic acid recovery system is imperative from a sustainability and economic viability perspective. The distillation sequence proposed above was developed considering distillation heuristics for favorable separations and economic operations. Within the second stage batch reaction, occurring within ‘BX2′, the bottoms stream from the primary distillation column, consisting of a mixture of acetin species only, is fed with the recovered acetic acid. Following this second phase reaction, complete selectivity to the desired higher esters (diacetin and triacetin) could be attained, with all the glycerol and monoacetin effectively converted. The product stream leaving the secondary batch reactor is fed into a final distillation column, whereby the desired product could be effectively isolated within the bottoms stream with high purity, with the excess acetic acid recovered within the distillate stream, which can be recycled and reused in subsequent batches.

The capital costs were estimated from the Aspen Process Economic Analyzer^®^ software (https://www.aspentech.com/). The analysis indicated that the capital costs were USD 71 M, while the operating costs were USD 303 M/year. The gross profit was USD 60.5 M/year, and the net present value (NPV) of the project was USD 235 M with a payback period of 1.7 years. A sensitivity analysis indicated that the product price has the most impact on the NPV.

The economic analysis performed by Keogh et al. revealed the process to be highly profitable [18] and thus definitively confirmed the commercial viability of the novel catalyst at an industrial manufacturing scale. The economic analysis has shown that the project could be highly profitable with an NPV of USD 235M for a project lifetime of 20 years. As shown by the sensitivity analysis, the project is stable as there are no major price changes predicted in the near future. 

## 3. Conclusions

A variety of acid catalysts have been shown to facilitate the production of glycerol esters through the esterification of glycerol with acetic acid. The use of acetic acid compared with acetic anhydride offers a more cost-effective and safer reaction pathway. To produce a higher selectivity toward di- and triacetin, a number of factors need to be considered. A higher selectivity can be facilitated through the use of a higher acetic acid to glycerol mole ratio. From a catalyst design perspective, a higher overall catalyst acidity results in better glycerol conversion and higher selectivity. Specifically, for solid catalysts, larger pore sizes facilitate the movement of the bulkier di- and tri-substituted products to and away from the catalyst active sites. From a scale-up and commercialization perspective, easy availability of catalysts at large scale, high stability, facile recovery, good recyclability, and low cost are key criteria. Based on these criteria, both SO_3_H-functionalized ionic liquids, for example [H-NMP][HSO_4_], and supported heteropoly acids, for example, tin-exchanged TPA supported on montmorillonite K-10 catalysts, are potential catalysts, with an excellent fit to the above catalyst design criteria. 

Ionic liquids are exciting homogeneous catalysts, with potential for customization to tailor the strength of acid sites, and they have good reusability. The ionic liquids reported for the esterification of glycerol with acetic acid have shown good activity and selectivity. However, the use of expensive components and multistep synthesis methods limits the industrial use of these catalysts. The development of more cost-effective acidic ionic liquid catalysts for the reaction should be pursued further.

Heteropolyacids have shown to be capable catalysts for the reaction. The use of support has been shown to be effective in catalyst heterogenization. Further work in this area should focus on the use of more acidic catalyst supports to increase the overall catalyst acidity. The effect of cost effectivity metal ion substitution to tailor the strength of acid site should also be considered.

The detailed techno-economic assessment and sensitivity analysis have shown the process to be highly profitable, thereby assertively confirming the economic viability of the glycerol esterification process at an industrial manufacturing scale.

## Figures and Tables

**Table 1:** Properties of glycerol [6,7].

Molecular weight	92.09 g mol^−1^
Density	1.25 g mL^−1^
Melting point	20 °C
Boiling point	290 °C
Flash point	160 °C
Autoignition point	393 °C
Viscosity (at 25 °C)	954 centipoises
pH	5.5–8

**Table 2:** Uses of glycerol acetin esters [43,44,45].

Monoacetin	Diacetin	Triacetin
Food additive	Oxygenate fuel additive	Oxygenate fuel additive
Manufacture of explosives	Plasticiser	Solvent
Smokeless powder	Softening agent	Food additive
Tanning agent	Solvent	Excipient of pharmaceutical products
Solvent for dyes		Plasticizer
		Antimicrobial and emulsifying agent in cigarette filters

**Table 4:** Conversion and selectivity for various ion-exchange resins [64].

Ion-Exchange Resin	Conversion of Acetic Acid (%)	Product Selectivity (%)			
		1-MA	2-MA	1,3-DA	1,2-DA
Amberlyst-15	95.3	63.4	7.0	2.0	0.5
Amberlyst-36	95.6	62.5	7.8	3.0	1.5
Dowex 50 WX2	95.2	71.9	8.8	3.3	1.7
Dowex 50 WX4	94.8	63.7	7.8	2.8	1.4
Dowex 50 WX8	94.7	64.9	8.1	3.1	1.5

Reaction conditions: 105 °C, 600 min, molar ratio of acetic acid to glycerol of 1:8, and 6.25 mg/mL catalyst.

**Table 5:** Comparison of Zr-modified mordenite [71].

Catalyst	Glycerol Conversion (%)	Product Selectivity (%)		
		MA	DA	TA
M	68.2	33.1	14.6	52.3
M1	89.3	19.3	17.0	63.7
Zr/M	74.4	32.2	24.6	43.2
Zr/M1	93.5	18.4	12.4	69.2

Reaction conditions: 100 °C, 180 min, molar ratio of acetic acid to glycerol of 10:1, and 5 wt% catalyst.

**Table 6:** Activity of silver-exchanged HPW in glycerol esterification with acetic acid [89].

Catalyst	Glycerol Conversion/%	Product Selectivity (%)		
		MA	DA	TA
HPW	70.3	59.3	37.7	3.0
Ag_1_PW	96.8	48.4	46.4	5.2
Ag_2_PW	82.5	52.7	43.4	3.9
Ag_3_PW	75.7	59.7	37.1	3.2

Reaction conditions: 120 °C, 15 min, molar ratio of acetic acid to glycerol of 10:1, and 1 wt% catalyst.

**Table 7:** Efficacy of different acid catalysts in the esterification of glycerol with acetic acid.

Catalyst	Operating Parameters				Performance	Ref.
	Temperature (°C)	Time (min)	Molar Ratio of Acetic Acid to Glycerol	Catalyst Loading		
Homogeneous Catalysts						
H_2_SO_4_	60	480	3:1	[H^+^] = 0.03 mmol	*C* = 98% *S* = 54% MA, 27% DA	[55]
p-TSA					*C* = 85% *S* = 86% MA, 8% DA	
1-butylpyridinium chloride—aluminium (III) chloride	75	120	3:1	1 mL	*C* = 100% *S* = 17.1% MA, 58.8% DA, 24.1% TA	[49]
[HSO_3_-pmim][HSO_4_]	120	360	8:1	6.25 mol%	*Y = 95.6% TA*	[50]
[(HSO_3_-p)_2_im][HSO_4_]	100	30	8:1	0.1 mol%	*C* = 95% *S* = 43.1% MA, 51.4% DA, 5.5% TA	[51]
PPS-TPA-HOAc	105	360	10:1	2.5 mol%	*C* = 100% *S* = 2.3% MA, 40.0% DA, 57.7% TA	[45]
[HO_3_S-(CH_2_)_3_-NEt_3_]Cl-[FeCl_3_]_0.67_	Reflux (Toluene)	240	5:1	0.3 mol%	*Y* = 98.6%	[53]
H_3_PW_12_O_40_	60	480	3:1	[H^+^] = 0.03 mmol	*C* = 96% *S* = 66% MA, 34% DA	[55]
H_4_SiW_12_O_40_	60	240	3:1	0.06 mol %	*C* = 100% *S* = 42% MA, 53% DA, 5% TA	[56]
Fe_4_(SiW_12_O_40_)_3_					*C* = 100% *S* = 24% MA, 69% DA, 7% TA	
Sn_1.5_PW_12_O_40_	70	180	12:1	0.78 mol%	*C* = 100% *S* = 4% MA, 56% DA, 40% TA	[57]
SnCl_2_.H_2_O	60	480	12:1	0.4 mmol	*C* = 96% *S* = 54% MA, 46% DA	[55]

## Data Availability

The data presented in this study are available in the article.
